# Hypoxia alters posterior cingulate cortex metabolism during a memory task: A ^1^H fMRS study

**DOI:** 10.1016/j.neuroimage.2022.119397

**Published:** 2022-06-23

**Authors:** Matthew Rogan, Alexander T. Friend, Gabriella MK Rossetti, Richard Edden, Mark Mikkelsen, Samuel J Oliver, Jamie H Macdonald, Paul G Mullins

**Affiliations:** a School of Human and Behavioural Sciences, Bangor University, Bangor, United Kingdom; b The Bangor Imaging Unit, Bangor University, Bangor, United Kingdom; c Institute for Applied Human Physiology, Bangor University, Bangor, United Kingdom; d Centre for Integrative Neuroscience and Neurodynamics, University of Reading, Reading, United Kingdom; e Russell H. Morgan Department of Radiology and Radiological Science, The Johns Hopkins University School of Medicine, Baltimore, MD, United States; f F. M. Kirby Research Center for Functional Brain Imaging, Kennedy Krieger Institute, Baltimore, MD, United States; g Department of Radiology, Weill Cornell Medicine, New York, NY, United States

**Keywords:** Hypoxia, Cerebral blood flow, Magnetic resonance spectroscopy, Posterior cingulate cortex

## Abstract

Environmental hypoxia (fraction of inspired oxygen (F_I_O_2_) ~ 0.120) is known to trigger a global increase in cerebral blood flow (CBF). However, regionally, a heterogeneous response is reported, particularly within the posterior cingulate cortex (PCC) where decreased CBF is found after two hours of hypoxic exposure. Furthermore, hypoxia reverses task-evoked BOLD signals within the PCC, and other regions of the default mode network, suggesting a reversal of neurovascular coupling. An alternative explanation is that the neural architecture supporting cognitive tasks is reorganised. Therefore, to confirm if this previous result is neural or vascular in origin, a measure of neural activity that is not haemodynamic-dependant is required.

To achieve this, we utilised functional magnetic resonance spectroscopy to probe the glutamate response to memory recall in the PCC during normoxia (F_I_O_2_ = 0.209) and after two hours of poikilocapnic hypoxia (F_I_O_2_ = 0.120). We also acquired ASL-based measures of CBF to confirm previous findings of reduced CBF within the PCC in hypoxia.

Consistent with previous findings, hypoxia induced a reduction in CBF within the PCC and other regions of the default mode network. Under normoxic conditions, memory recall was associated with an 8% increase in PCC glutamate compared to rest (*P* = 0.019); a change which was not observed during hypoxia. However, exploratory analysis of other neurometabolites showed that PCC glucose was reduced during hypoxia compared to normoxia both at rest (*P* = 0.039) and during the task (*P* = 0.046).

We conclude that hypoxia alters the activity-induced increase in glutamate, which may reflect a reduction in oxidative metabolism within the PCC. The reduction in glucose in hypoxia reflects continued metabolism, presumably by non-oxidative means, without replacement of glucose due to reduced CBF.

## 1. Introduction

The human brain is in a continuous metabolically active state. To precisely serve this persistent energetic demand a multicellular orchestra between the neurons, glia and the vasculature has developed to attune the cerebral blood flow (CBF) delivery to metabolic demand (Duncombe et al., 2017) and neural activity. This neurovascular coupling is imperative for neuronal survival and sustaining optimal brain function. Environmental hypoxia, a reduction in oxygen availability, provides an eloquent model for understanding the perseverance of this coupled relationship during physiological stress.

Upon exposure to hypoxia, global CBF (gCBF) increases concomitantly with falling arterial oxygen saturations (Ainslie and Subudhi, 2014). The increase in gCBF is hypothesised to maintain the cerebral oxygen delivery sustaining the global cerebral metabolic rate of oxygen (CMRO_2_) (Ainslie et al., 2014). However, regional measurements have revealed heterogeneous regional CBF increases and decreases in response to acute hypoxia ranging from minutes to hours (Lawley et al., 2017; Nöth et al., 2008; Rossetti et al. 2020). In particular, regions in the posterior of the brain that are known to constitute major nodes in the default mode network (DMN), the posterior cingulate cortex (PCC) and angular gyri, were hypoperfused during hypoxia (Lawley et al., 2017; Rossetti et al., 2020). This finding of reduced blood flow may reflect altered metabolism in these regions, even though global metabolism is maintained.

When measured globally, cerebral metabolism has been found to increase (Smith et al., 2013; Vestergaard et al., 2016; Wang et al., 2015), decrease (Jensen et al., 2018) or be unchanged (Vestergaard and Larsson, 2019) during periods of acute poikilocapnic hypoxia. Regional investigations of cerebral metabolism are less common and have utilised variations in intensities and durations of hypoxia. Of such investigations, Hochachka et al. (1996) demonstrated that whole brain, and regional, cerebral glucose metabolism in Tibetan high-altitude natives did not significantly differ from that observed in lowlanders using 2-[^18^F]deoxy-2-fluoro-D-glucose positron-emission tomography (^18^FDG-PET). However, the phylogenetically younger, less well adapted, high altitude dwelling Quechua natives, displayed a near uniform reduction in regional cerebral metabolism compared to lowlanders and Tibetan high-altitude natives (Hochachka et al., 1996). Furthermore, lowlanders who travel to high altitude have demonstrated both reductions and increases in regional cerebral metabolism (Hochachka et al., 1999; Merz et al., 2006). The authors concluded that the reduction in regional cerebral metabolism may serve as a defence mechanism in the face of hypoxia, akin to that observed in hypoxia-adapted vertebrates (Hochachka et al., 1996, 1999). More recently, magnetic resonance spectroscopy (MRS) has been used to assess the neurochemical profile in response to acute exposure to poikilocapnic hypoxia. Cerebral lactate concentrations have been found to increase in response to hypoxia (Edden et al., 2010; Jensen et al., 2018; Vestergaard et al., 2016; Vestergaard and Larsson, 2019) suggesting an increase in anaerobic glycolysis. This lactate is not a result of increased lactate in the blood, and instead likely reflects production within the brain tissue itself (Harris et al., 2013). Furthermore, occipital lobe glutamate concentration was found to increase along with global measures of CMRO_2_ (Vestergaard et al., 2016), suggestive of increases in metabolic activity. This finding was not replicated in a follow-up study, with the authors citing the individual responses to poikilocapnic hypoxia and the subsequent vascular and metabolic alterations involved with the superimposed chemostimuli of hypoxaemia and hypocapnia (Vestergaard and Larsson, 2019).

Although earlier work that suggested metabolic suppression as a defence mechanism in the face of hypoxia (Hochachka et al., 1996; Hochachka et al., 1999) could provide an explanation for the regional perfusion reductions reported (Lawley et al., 2017; Nöth et al., 2008; Rossetti et al., 2020), regional investigations using MRS during acute hypoxic exposure currently do not support a reduction in resting metabolic activity (Vestergaard et al., 2016; Vestergaard and Larsson, 2019). Therefore, alternative explanations, such as hypoxia inducing a change in vascular signalling mechanism within the PCC, that either overrides typical neurovascular coupling or reverses it (Rossetti et al., 2020) require further investigation. These previous findings suggest the response of regional cerebral vascular coupling to hypoxia is more nuanced than observed globally.

Without directly probing metabolism in the brain regions undergoing reductions in blood flow during hypoxia, it is unclear whether the brain is suppressing neural activity and metabolic rate during hypoxia to ensure oxygen supply suffices demand, or if there is an alteration in the standard coupled relationship. To further investigate this possible change in metabolic coupling, a recent investigation by Rossetti et al. (2020) utilising a memory association task, revealed an alteration in the task-induced BOLD responses during hypoxia. Specifically, regions that during normoxia showed a positive BOLD signal in response to the task reversed, showing a negative BOLD signal in hypoxia, and the opposite also happened – whereby a task induced negative BOLD in normoxia became a positive BOLD response in hypoxia (Rossetti et al., 2020). Generally, negative BOLD measures are interpreted to suggest regions where neural activity has been actively suppressed. However, reversal of the BOLD response to task-based neural activation has been observed in the infant brain (Kusaka et al., 2004; Yamada et al., 2000). Neonatal rat models indicate this infantile reversal in BOLD response to task results from insufficient hyperemia in response to neural activity and post-stimulus pial vasoconstriction (Kozberg et al., 2013; Kozberg and Hillman, 2016). Indicating that the mechanisms of the negative BOLD response may depend on the specific temporal characteristics and metabolic conditions involved. The findings of Rossetti et al. (2020) suggest hypoxia is one such plausible metabolic condition. The unusual haemodynamic response in these conditions is hypothesised to be the result of neural activation stimulating local vasoconstriction, impeding CBF delivery, in a reversal of standard neurovascular coupling. Nevertheless, as the study of Rossetti study did not utilise any additional, non-vascular related techniques as a measure of metabolism or neural activity in conjunction with the BOLD signal, it is difficult to conclusively disentangle vascular effects from neural activity, and so this phenomenon requires further investigation.

Adapting static MRS to allow for high-frequency repeated acquisitions in the order of seconds provides a method to probe the dynamic changes in neurochemicals during functional activation. This functional MRS (fMRS) technique has been used to measure the functional dynamics of the neurotransmitter glutamate to a variety of tasks (Mullins, 2018). The measured functional change in neurotransmitters has also been found to regionally correlate with the direction of change in the BOLD-weighted MRI contrast (Bednařík et al., 2015; Boillat et al., 2020; Ip et al., 2017, 2019; Martínez-Maestro, Labadie and Möller, 2018), serving as a complimentary measure of neural activity. Critically, this technique provides a measurement of neural activity that unlike the BOLD signal, is not haemodynamically-dependant. Thus, when alterations in neurovascular coupling are suspected, fMRS serves as a reliable and direct measurement of neural activity (Stanley and Raz, 2018).

The current experiment employed an event-related fMRS protocol during a paired associate memory recall task to measure task-related glutamate dynamics during normoxia and hypoxia. This investigation allowed the probing of task-related neural activity in the PCC independent of haemodynamic responses. In doing this we aimed to understand if the observation of Rossetti et al. (2020) during exposure to environmental hypoxia is the result of a reversal in neurovascular coupling or suppression of neural activity during hypoxia. Our hypothesis therefore was twofold. 1: In response to a memory recall task, glutamate concentration within the PCC will increase during normoxia. 2. Hypoxia will not change this task-induced glutamate response. A lack of a task induced glutamate response in hypoxia however would suggest that hypoxia alters the typical neuronal response, and may be one of the reasons for the change in CBF previously reported.

## 2. Methods

### 2.1. Participants

Fifteen healthy adults (5 females) were recruited into the study ((mean ± SD); age, 25 ± 4 years; height, 177 ± 10 cm; body mass, 77.3 ± 12.3 kg). Participants had not traveled to altitude (> 1500 m) in the preceding six months and had no medical contraindications. Female participants were studied during the early follicular phase of their cycle, or the placebo phase of oral contraceptives. All participants provided written informed consent. Ethical approval was granted by the Ethics Committee of the School of Psychology at Bangor University, and carried out in accordance with the WMA Declaration of Helsinki. All scanning procedures were scrutinized and approved by the Bangor Imaging Unit steering committee. (Ethical approval number 2019–16489).

### 2.2. Study design

The study followed a repeated-measures, counterbalanced cross-over design. See Supplementary Fig. 1 for a design and procedure schematic. Experimental sessions were separated by at least five days and the procedures in each session were exactly matched with all physiological data collected covertly so participants would not detect which condition they were in. Participants completed an encoding and familiarization session for the paired associate memory task the day before each experimental session. Experimental sessions consisted of 3.5 h exposure to normoxia (fraction of inspired oxygen; F_I_O_2_ = 0.209) or hypoxia (F_I_O_2_ = 0.120). At the 2 h time point, MRI was commenced allowing *T*_1_-weighted structural images, arterial spin labelling (ASL) measures of resting perfusion, and fMRS to measure task-induced changes in neurochemicals detectable in the ^1^H spectrum during a paired associate memory task, all to be obtained.

### 2.3. Experimental protocol

All procedures were performed within 50 m of sea level. Participants completed the first 2 h of each session in a temperature and humidity controlled environmental chamber (Hypoxico Inc; NY), and the final 1.5 h of each session in a 3T MRI scanner. Throughout transportation between the chamber to the MRI suite and subsequent MRI scanning session, participants wore a leak-free face mask connected to a two-way Hans Rudolph valve with an inspiratory port connected via Falconia tubing to a 1000 L Douglas bag containing F_I_O_2_ = 0.209 or F_I_O_2_ = 0.120 (dependent on session condition).

### 2.4. Physiological monitoring

Physiological monitoring was conducted throughout the experiment. Heart rate and oxygen saturation (S_p_O_2_) were measured at thirty-minute intervals for the first two hours. Heart rate was measured using a 3-lead electrocardiogram (ECG) (Acuson X300, Siemens Healthcare GmbH; Erlangen: Germany), and S_p_O_2_ was measured using pulse oximetry (9550 OnyxII; Nonin Medical Inc, Minnesota). Expired carbon dioxide (CO_2_) was sampled from the face mask for a five-minute interval at 2, 2.5 and 3 h time points when in the MRI scanner. CO_2_ concentrations were estimated using a calibrated fast responding gas analyzer (GC-0017 (0–20%) SprintIR CO_2_ Sensor; GSS, Cumbernauld, UK), and recorded using CO_2_ logging software (GasLab; CO_2_Meter, Inc.; Florida, USA). The partial pressure of end-tidal CO_2_ (P_ET_CO_2_) was calculated from the recorded CO_2_ trace using peak detection software within the pracma package (Version 2.3.3, Borchers, 2018) in RStudio (Version 1.3.1073; RStudio Team, 2020).

Exposing humans to an acute hypoxic environment can cause acute mountain sickness (AMS) symptoms. To track the development of this condition participants were administered the Lake Louise Questionnaire (LLQ) (Roach et al., 2018) at thirty-minute intervals throughout the experimental protocol. Clinical AMS was defined as scoring ≥ 3 on the LLQ with the presence of headache and at least one other symptom. The presence of clinical AMS during the protocol was an exclusion criterion.

### 2.5. Memory task

The paired associate memory task paradigms were written in Octave, using PsychToolbox 3 (Brainard, 1997; Pelli, 1997) and has been reported previously in Rossetti et al., (2020) however the procedure is briefly explained below.

The day before each experimental session, participants completed an encoding session, where they were presented with two lists of images each containing 50 associate pairs of items taken from the Rossion and Pourtois pictorial set (Rossion & Pourtois, 2004), and were asked to commit the pairs to memory. Each list consisted of 25 semantically-related pairs, and 25 semantically-unrelated pairs. Images were presented for 1.5 s each, separated by 0.5 s. To ensure task engagement, participants were asked to provide a rating of the relatedness of the items in each pair on a four-item scale, ranging from “extremely unrelated” to “extremely related”. Items were repeated across the two lists within each session but were not repeated across the two encoding sessions (one for each experimental session). To enhance learning, participants completed a two-alternative forced choice (2AFC) recall task immediately after studying each list.

During the experimental session, participants were presented with a cue image (for 1.5 s) immediately followed by a second image (for 1.5 s) that was either the associate pair (target) or not (foil). The participant was asked to determine whether the cue and the second image were paired associates. Participants were encouraged to prioritize accuracy over speed. They provided a yes/no judgement as well as rated their confidence (high/low). In each experimental session, participants completed a total of 100 trials, split over 4 runs of 25 trials each, with jittered inter-trial intervals of 4, 6 or 8 s.

To control for any potential order effects on performance of the memory recall task, the order of the test conditions (normoxia or hypoxia) was counterbalanced across the participants, with 9 experiencing normoxia first and hypoxia second, and 6 experiencing hypoxia first, and normoxia second. We tested the data for an order effect on performance (paired T-Test comparing day of test) and found no effect of order.

### 2.6. Anatomical MRI

All MRI sequences were conducted on a 3T Philips Achieva MRI scanner (Philips Healthcare) using a 32-channel head coil. Anatomical image scans were acquired at the beginning of each scan protocol, immediately after a brief survey scan in normoxia and hypoxia experimental sessions. High resolution *T*_1_-weighted images were acquired as a five-echo MP-RAGE sequence (TE = 3.5, 10.5, 20.5, 30.5, 40.5 ms; TR = 45 ms, TI = 1150 ms; 3D acquisition; field-of-view = 225 mm × 225 mm × 175 mm; voxel dimensions = 1 × 1 × 1 mm^3^, SENSE = 2). The five echoes were then averaged to produce a single image used for registration of the arterial spin labelling scans.

### 2.7. Resting ASL perfusion

To measure whole brain resting perfusion, ASL images were acquired before the fMRS memory task in normoxia and hypoxia. ASL images were acquired using the standard single-phase pulsed ASL package provided with the scanner. Labeling of inflowing blood was achieved through a parallel slab applied 20 mm below the acquisition slices (slab thickness = 100 mm, post label delay = 1600 ms, SENSE = 2) without background suppression or use of a QUIPSS II modification to temporally define the bolus duration. We used a sagittal phase contrast angiographic image to plan the ASL image acquisition and labelling slice location, such that the labelling slice was in the straight part of the carotids. Post labelling delay was selected based on that used in the previous studies in our lab (Lawley et al., 2016; Rossetti et al., 2020). Each scan consisted of 22 slices with 256 × 256 mm^2^ field-of-view and 2 × 2 × 6 mm^3^ in plane resolution. All slices were aligned perpendicular to the Z-axis of the scanner. Slices were acquired as one tagged and one control, each acquired with 40 averages, with a TR of 3 s, and TE of 15 ms, giving a scan time of ~4 min. Image analysis was performed using the FMRIB Software Library (FSL) v6.0.1. MP-RAGE images were brain extracted using BET (Smith, 2002) then segmented using FAST (Zhang et al., 2001). ASL data were analysed using BASIL (Chappell et al., 2008). As ASL signal is dependent on blood T_1_, and as blood T_1_ values are dependent on haematocrit and blood oxygenation, we corrected the blood *T*_1_ estimates used in BASIL via the model of Hales et al. (2016) for each participant using their haematocrit and SpO_2_ values for each imaging session. Haematocrit, SpO_2_ and calculated T1 values used for the ASL processing can be found in supplementary table S2. Automatic estimation of bolus duration was applied. The CBF maps produced by BASIL were registered to the *T*_1_-weighted structural images, smoothed with a 4 mm Gaussian kernel, masked with the grey matter image from the *T*_1_ segmentation, and registered to the Montreal Neurological Institute (MNI) 2 mm *T*_1_-weighted average image for group comparison.

### 2.8. Static ^1^H MRS and ^1^H fMRS acquisitions

Both the fMRS and static MRS acquisition voxel were positioned over the posterior portion of the cingulate gyrus. Static MRS refers to spectra acquired separate to, and before the start of, the fMRS runs whilst the participant was rested staring at a fixation cross for the acquisition duration. Unless the participant reported movement or was moved, the location of the voxels stayed the same across the static MRS acquisition and all four fMRS runs. The average location of this voxel across fMRS runs, participants and conditions (normoxia and hypoxia) is shown in Fig. 2. Separately, the average location of the static MRS voxel across participants and conditions is shown in supplementary material Fig. 2. All spectra were acquired using a single-voxel PRESS sequence with a voxel size of 20 × 20 × 20 mm^3^, TE = 40 ms, TR= 2 s, comprising of 2048 data points and a spectral width of 2000 Hz, with CHESS water suppression. A PRESS sequence with a TE of 40 ms was chosen as this has previously been shown to provide reliable measures of Glutamate at 3T (Mullins et al 2008). Each fMRS run lasted for approximately 5 min and 30 s acquiring 168 shots, whilst the static MRS was shorter lasting approximately 2 min and 40 s acquiring one spectrum formed of 64 averages. A reference water scan, using the same PRESS sequence, but with water suppression off, and 16 averages, was acquired before each fMRS run. For the fMRS runs the standard acquisition sequence was modified, allowing the scanner to send a transistor–transistor logic (TTL) pulse upon each acquisition for the duration of the scan. The acquisitions were acquired as individual “shots”, producing a separate free induction decay signal (FID) for each shot. The beginning of each trial was triggered 1700 ms after the first TTL pulse. This allowed the precise presentation of the cue image 300 ms before the acquisition of the first shot in a trial period, then shots were acquired every 2 s after until the trial ended (participants response registered). The end of the trial was followed by a randomized ITI of 4, 6 or 8 s. The beginning of the next trial was triggered again by the TTL pulse, ensuring shots in all trials were time-locked to the same acquisition timeline. See Supplementary Fig. S1 for a schematic of the MRS acquisition protocol. A summary of the MRS and fMRS details is also included following the MRSinMRS checklist (Lin et al., 2021) as supplementary table S3.

#### 2.8.1. Analysis of metabolite and fMRS spectra

**Fig. 1.**
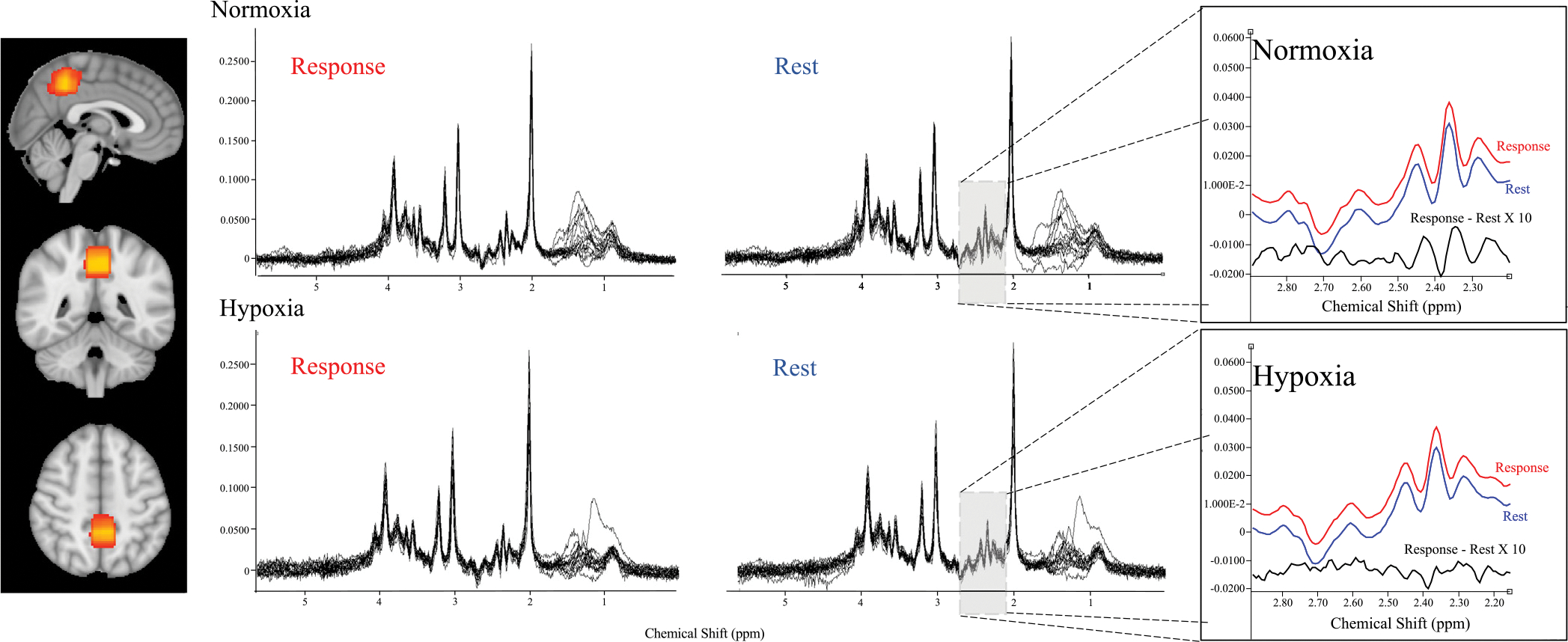
The average location of the MRS acquisition voxel is shown on the left. Yellow reflects greater overlap in positioning across participants and conditions. Acquired spectra across all participants in each condition are shown for visual assessment of quality. On the right is the result of the subtraction (magnified × 10) of rest from response spectra averaged across all participants for each condition, showing a glutamate increase in the normoxia condition.

The reliability of the MRS signal is dependent on the number of FIDs within the averaged spectrum. Dividing spectra based upon response type (such as only using correctly recalled trials) within the task would have a detrimental effect on the signal-to-noise ratio (SNR) of the average spectrum and the estimated concentration of metabolites (Lally et al., 2014), therefore all trials (hits and misses) were included in the recall condition average. To boost the SNR, average spectra were created by binning shots within task types across all 4 runs. Each run consisted of 25 trials. Rest was always the shot acquired before the beginning of a trial, where an individual would be viewing a blank screen with a single central fixation dot. These shots correspond to the period between the latter part of the intertrial interval just before the triggering of the next trial (always 100 FIDs). Recall corresponds to the FID acquired between the presentation of the second image and the time at which the participant makes their response (providing at least 100 FIDs for the response period, when averaged across all runs). This analysis setup allowed the greatest number of shots to be averaged into the rest and recall spectra, providing sufficient SNR for subsequent metabolite estimation and comparison between spectra for each participant in each condition. Fig. 1 gives a schematic representation of how spectra are assigned to either rest, or response before binning to produce an average spectrum.

The fMRS spectra (see Fig. 1 for example) and static MRS spectrum (see Supplementary Fig. 2 for example) were processed and analysed using the Java-based version of the magnetic resonance user interface (jMRUI; (Naressi et al., 2001); software version 6.1 (http://www.jmrui.eu)). Signal amplitudes were estimated using quantification based on quantum estimation (QUEST) and absolute concentrations were produced by referencing to an unsuppressed water peak acquired immediately before the acquisition of the static MRS and each fMRS run. The metabolite basis set included 19 metabolites including acetate, aspartate, choline, creatine, cysteine, gamma-aminobutyric acid, glucose, glutamate, glutamine, glutathione, glycine, lactate, myo-inositol, *N*-acetylaspartate, *N*-acetylaspartylglutamate, phosphorycholine, scyllo-inositol, taurine and valine. The baseline subtraction protocol within jMRUI’s QUEST quantitation tool was used to handle the macromolecule baseline in both the fMRS and static MRS (Stefan et al., 2009).

### 2.9. Statistical analysis

#### 2.9.1. Perfusion comparisons between conditions

A paired-samples *t*-test for whole-brain CBF changes between normoxia and hypoxia was performed in RANDOMISE (Winkler et al., 2014) (cluster mass FWE correction at *p* < 0.05). Furthermore, using the average position of the MRS voxel across participants in each condition, we extracted the mean perfusion of this region and compared between conditions.

#### 2.9.2. Analysis of behavioural performance

All statistical comparisons were conducted in RStudio (Version 1.3.1073; RStudio Team, 2020) utilising the tidyverse package (version 1.3.0; Wickham et al., 2019). Responses to the task were categorised as either hit, miss, correct rejection or false alarm. Performance was calculated as the proportion of hit and correct rejection trials to miss and false alarm trials. Average reaction time (RT) across runs for each condition (normoxia and hypoxia) was compared using paired *t*-test to assess for the presence of a speed accuracy trade off. We calculated the signal detection theory indices dprime (D’) and beta using the ‘dprime and other signal detection theory indices’ function within the psycho package (Version 0.5.0; Makowski, 2018). D’ is a measure of discriminability from chance in performance. It was calculated from the proportion of hits and false alarms. The beta attempts to summarise the response bias across the trials in each separate condition.

#### 2.9.3. Statistical comparison of metabolite concentrations

To test the two-part hypothesis, a two-by-two repeated measures analysis of variance (ANOVA) was used to assess whether glutamate concentrations within the PCC showed task-related increases during the paired associated memory task and if the presence of hypoxia did or did not alter this. From this analysis, post hoc paired t-tests were undertaken to explore any significant main effects between the task state (rest vs recall) and condition (normoxia and hypoxia). Metabolite concentrations acquired using the static MRS in normoxia and hypoxia were compared using a paired samples t-test. All statistical comparisons were carried out in RStudio (Version 1.3.1073; RStudio Team, 2020) utilising the tidyverse package (Version 1.3.0; Wickham et al., 2019) and rstatix package (version 0.6.0; Kassambara, 2020).

## 3. Results

### 3.1. Hypoxia increased heart rate while decreasing arterial oxygen saturation and end-tidal CO_2_

Compared to normoxia, 2 h of poikilocapnic hypoxia increased heart rate by 6 bpm (95% CI: [12, 0]; *P* = 0.077) and reduced peripheral arterial oxygen saturation by 16% (95%CI: [− 18, −11]; *P* < 0.001). See supplementary table S1 for mean data.

P_ET_ CO_2_ was reduced in hypoxia compared to normoxia by 2 mmHg (95% CI: [−5, 0]; *P* = 0.169) at the 2 h time point. By the second measurement at the 2.5 h time point it had reduced further being 5 mmHg lower than that measured during normoxia (95% CI: [−8, −2]; *P* = 0.051). Upon the final measurement at 3 h it appeared to stabilize at an average 5 mmHg reduction (95% CI: [−9, −1]; *P* = 0.098). See Supplementary Table S1 for mean data.

Comparing participant average LLQ score across each condition revealed no significant differences between normoxia (mean ± SD; 0.22 ± 0.43) and hypoxia (0.59 ± 0.65, *P* = 0.123).

### 3.2. Hypoxia altered resting cerebral perfusion in a regional manner

Cerebral perfusion was analysed in 13 of the 15 participants. This was because in one of the experimental conditions, the scan was not collected due to termination of the session before ASL collection: both individuals reported feelings of discomfort (symptoms of AMS) that surpassed our predefined threshold. Due to the paired nature of the data, lack of data in one condition results in exclusion from the data analysis.

**Fig. 2.**
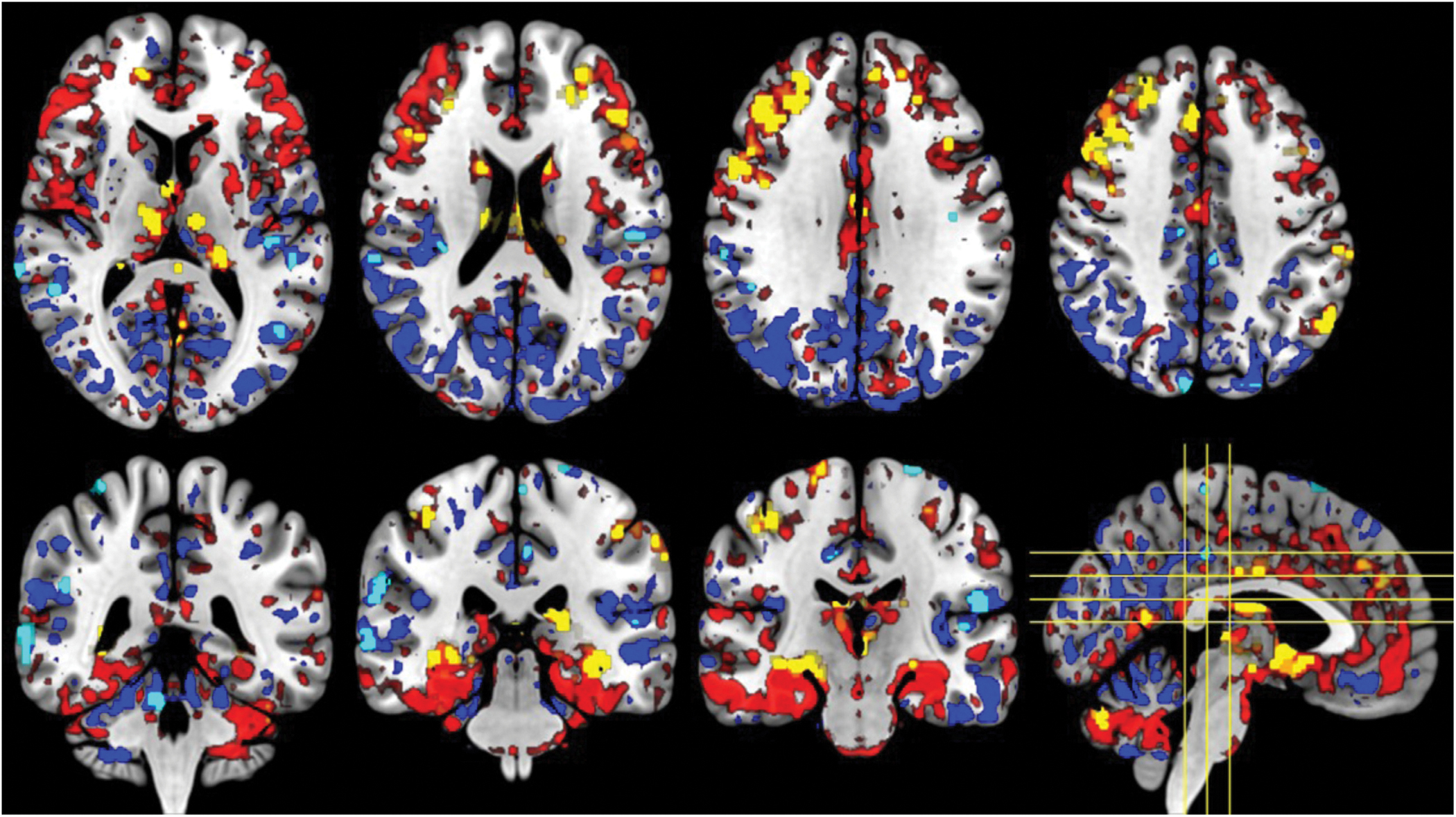
Significant clusters of reduced (light blue) and increased (yellow) regional CBF during hypoxia overlayed with absolute perfusion increases (red) and decreases (darker blue). Clusters were calculated using FSL tool RANDOMISE with cluster-based thresholding and FWE correct set at *p* < 0.05

Significant clusters of reduced perfusion during hypoxia compared to normoxia were revealed within the PCC and wider posterior regions (see Fig. 2). Clusters of increased perfusion during hypoxia were identified also, however these tended to reside within the anterior regions of the brain.

A region of interest analysis using the mean MRS voxel location as a mask revealed a nonsignificant 15ml/100 g/min reduction in perfusion in hypoxia compared to normoxia (95% CI: [−73, 43]; *P* = 0.499).

### 3.3. Hypoxia did not alter neurochemistry within the PCC at rest

Analysis of the resting neurochemistry of the PCC from the static MRS acquisition was formed of 11 data sets as it was never acquired in 4 of the participants. Exposure to 2.5 h of acute poikilocapnic hypoxia did not alter the concentration of any measured metabolite within the PCC at rest. For the estimated metabolite concentrations and between condition comparisons, see Table 1.

### 3.4. Hypoxia was detrimental to task performance

A summary of the participants average performance can be found in Table 2. After 2.5 h, hypoxia significantly reduced participant memory recall accuracy by 8% (95% CI: [−13, −2]; *P* = 0.012). However, reaction time was unaffected (*P* = 0.502).

To understand the change in accuracy we calculated the D’. A higher value of D’ indicates better performance than chance. This value, alongside performance, was found to be significantly reduced by 28% (95% CI: [−50, −6]; *P* = 0.016) in hypoxia compared to normoxia. We found no significant alteration in beta between the two conditions (*P* = 0.305), which suggests the observed change in performance was not the result of a response bias.

### 3.5. Hypoxia altered task-induced neural activity/metabolism within the PCC

Table 3 displays the concentration estimations for the rest and response in both normoxia and hypoxia. Across all participants, metabolite estimation for Glutamate, Myo-Inositol, Creatine, N-Acetyl Aspartate, and Choline, had a standard deviation lower than 10%. Glutamine, Glucose and Glutathione had a SD lower than 40%. Lactate and Gamma-Aminobutyric Acid had a SD greater than 40%. The full width at half maximum (FWHM) for the NAA peak (Hz) in normoxia for rest (mean ± SD; 4.74 ± 2.33) and response (4.60 ± 2.64) did not significantly differ (*P* = 0.464), nor did it for rest (5.67 ± 2.71) and response (5.74 ± 2.63) in hypoxia (*P*=0.609).

**Fig. 3.**
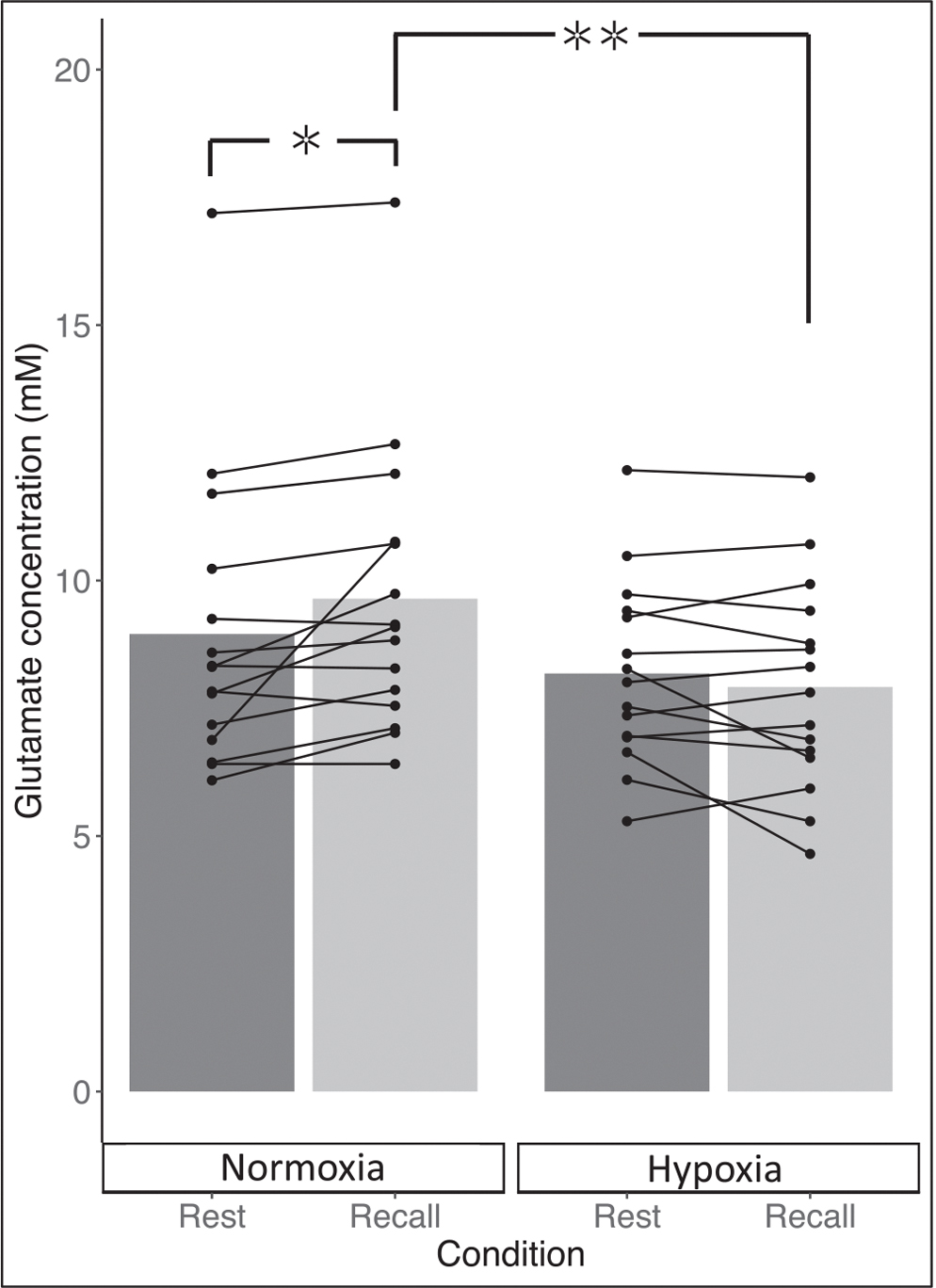
Glutamate metabolite concentration (mM) during rest (grey) and memory recall during the task (light grey) in both normoxia (left) and hypoxia (right). Connected lines represent individual participant change within condition between rest and recall. ✲ denotes significant difference at *P* < .05, ✲ ✲ *P* < .001

**Fig. 4.**
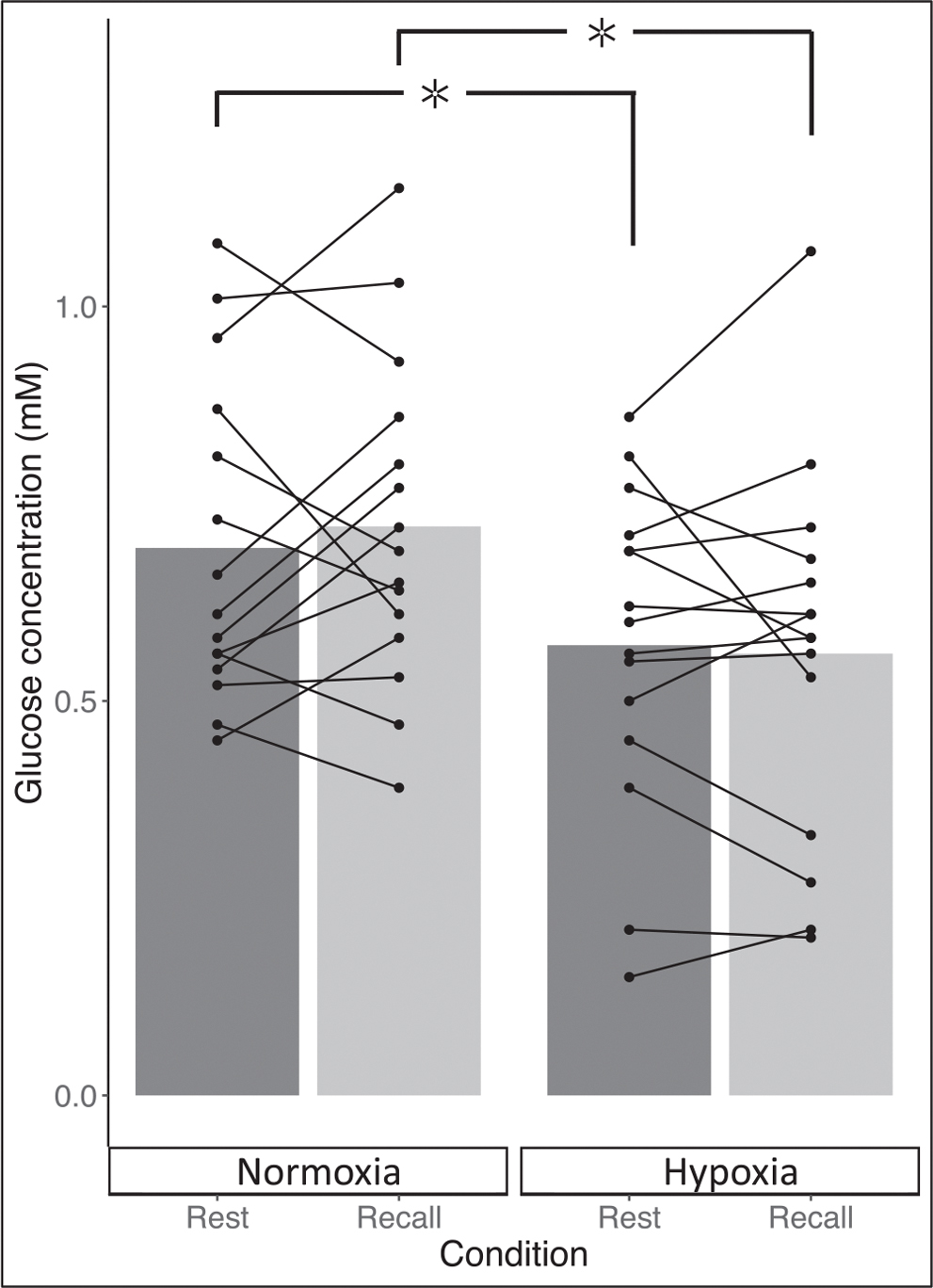
Glucose metabolite concentration (mM) during rest (grey) and memory recall during the task (light grey) in both normoxia (left) and hypoxia (right). Connected lines represent individual participant change within condition between rest and recall. ✲ denotes significant difference at *P* < .05, ✲ ✲ *P* < .001.

In line with the first part of our hypothesis glutamate increased by 8% between rest and memory recall in normoxia (95% CI: [0, 15]; *P* = 0.019; Fig. 3). This task-induced increase was not present in hypoxia: participants showed a non-significant reduction of 3% (95%CI: [CI −9, 2]; *P* = 0.219; Fig. 4).

Further exploratory analyses revealed glucose concentration was lower during both rest and recall in hypoxia compared to normoxia. Specifically, glucose concentration was 18% lower during rest (95% CI: [−34, −2]; *P* = 0.046; Fig. 4) and 23% lower during recall (95% CI: [−45, 0.5], *P* = 0.039; Fig. 5). Glutathione concentration during recall was also found to be 28% lower during hypoxia compared to normoxia (95% CI: [−47, −9]; *P* = 0.048; Table 3).

## 4. Discussion

This study used fMRS to measure the metabolite dynamics of the PCC during episodic memory recall in both normoxia and hypoxia, as well as ASL to measure CBF changes. As initially hypothesised, we showed that memory recall results in a significant increase in glutamate concentration within the PCC. However, this task-induced change in glutamate concentration was not observed during hypoxia, suggesting a reduction in activity within the PCC to this cognitive task during hypoxia. Consistent with this neurochemical observation, a reduction in participant memory recall accuracy was also observed, during hypoxia. However, a reduction in glucose was observed during hypoxia, suggesting increased or maintained glycolytic metabolism. These results show that hypoxia impacts both CBF and neural responses, and may induce a shift from oxidative metabolism towards anaerobic glycolysis.

It is assumed that the regionally-specific metabolic demand for oxygen associated with resting and functional neurotransmission is met by a pervasive coupling with the moment-to-moment distribution of regional CBF. This neurovascular coupling is vital for sustaining neural health and is the assumption that underlies functional magnetic resonance imaging (Logothetis, 2008). Hypoxia, in reducing arterial oxygen tension and thus the availability of oxygen to the cerebral tissue, challenges this coupled relationship. Exposure to hypoxia has demonstrated a non-uniform regional cerebral blood flow response at the level of the extracranial arteries (Binks et al., 2008; Willie et al., 2014) and downstream heterogeneity in regional cerebral perfusion has been observed (Lawley et al., 2017; Nöth et al., 2008; Rossetti et al., 2020).

We interpret the ASL results of the present study to confirm two previous findings of consistent regional perfusion reductions in the posterior portion of the cingulate gyrus (PCC) in response to hypoxia (Lawley et al., 2017; Rossetti et al., 2020). However, for completeness due to the nature of the ASL sequence used there is another possible explanation, that of a regional change in the arterial transit time (ATT), with ATT either decreasing, or increasing in a region-specific manner. An increase, or delay in ATT to the PCC, would produce a reduced ASL signal, and would be entirely consistent with a reduction in blood flow, as we interpret the data. However, a reduction in ATT (shorter transit time), with concomitant increased CBF, might mimic a loss, as tagged blood may arrive and leave the regions of interest before the ASL tag image is acquired, leading to a reduced ASL signal. This would not agree with our interpretation of a reduction in CBF, instead supporting an increase in CBF. However, we feel there are three arguments against a shorter ATT being behind our results in this (and previous studies): (1). ASL imaging of perfusion does not just measure tagged water within the blood in the arteries and arterioles as it arrives, it also measures tagged water within blood in the capillaries, as well as tagged water that has exchanged from the blood into the tissue itself. In fact, the delay time is often chosen to weight more towards the tagged water in the microcirculation which has perfused into the tissue, so a shorter ATT should not have a large effect on our results.; (2). ASL using a single delay time is not that sensitive to reduced ATT, with the tagged ASL signal being more dependent on the T1 decay of the tagged water in the capillaries and in the tissue than arterial supply. Indeed, a faster ATT is considered beneficial in single delay ASL as it reduces the influence of arterial blood on the ASL signal, allowing a better estimate of perfusion (Alsop et al., 2015), and it is only excessively long ATT that causes a problem (see previous point about an increased or slower ATT).; and (3) In previous work by Lawley et al (2017), a 5% hypercapnic challenge at the 2 hr time point in hypoxia demonstrated that only those regions which had shown a reduction in CBF as a result of hypoxia showed a significant increase in CBF in response to the hypercapnic challenge. These were the same regions we report here. Given the well documented effect of a hypercapnic challenge as a vasodilator and potent enhancer of CBF, it seems logical that these regions where the only ones to respond significantly because they were the only ones that had the capacity to do so to a significant extent. Other regions did not have capacity to vasodilate as they had already dilated in response to hypoxia (as evidenced by increases in CBF). The PCC and angular gyri, however, were able to vasodilate in response to CO_2_ because the vasculature was more constricted in hypoxia, meaning there was more capacity to dilate. We therefore feel that all of these arguments strongly support that fact that we are indeed measuring a reduction in CBF with our experiment, and not just a change in ATT.

The origins of this previously reported reduction in cerebral perfusion during hypoxia had two possible interpretations. The first assumed a coupling between neural activity in those regions and CBF delivery, concluding that the region has reduced its metabolic demand. This would align with early observations of metabolic suppression in high altitude adapted humans (Hochachka et al., 1996; Hochachka et al., 1999). However, an alteration in neurovascular coupling cannot be ruled out. Recent findings by Rossetti et al (2020) support this, demonstrating that during functional activation of the PCC, the BOLD signal response was reversed in hypoxia (a positive response became negative, and importantly a negative response became positive), without any change in performance. This observation suggested a reversal in the normal mechanism of neurovascular coupling within the PCC (Rossetti et al., 2020). However, without an independent measure of neural metabolism in that region, that conclusion remained speculative.

We measured the neurometabolic profile of the PCC at rest, using static MRS after 2.5 h of hypoxia. We did not reveal any significant alteration in the resting neurometabolism within this region in a static measure (baseline MRS) despite the observed regional alteration in perfusion in our study. We did, however, find a dynamic change in glutamate and glucose levels in a task-based fMRS paradigm. This static or baseline finding adds to results from previous studies that have recorded both increases and no change in regional glutamate concentration (Vestergaard et al., 2016; Vestergaard and Larsson, 2019) at rest during exposure to acute hypoxia. We tentatively conclude that with a reduction in perfusion and given the state of hypoxia, there is a reduction in the delivery of oxygen to the cerebral tissue, yet it is still enough to maintain “resting” metabolic activity. However, it is important to note, that the average MRS spectrum used to quantify neurometabolites was acquired over a large 8 mL region of cerebral tissue, whilst perfusion measures are localised to 0.024 mL voxels. As a result, a more spatially resolved measurement of neurometabolites may be warranted to unveil the precise topology of resting CBF and neurometabolic alterations between normoxia and hypoxia. We also utilised a PRESS sequence that was not optimised for the detection of some lower concentration metabolites such as lactate, which if measured reliably could be critical in the interpretation of the resting metabolic profile of this region.

Event-related fMRS sequences provide a novel and flexible technique to study functional alterations in neurotransmitter systems and metabolites. In employing this novel methodology, the present study reports an 8% increase in glutamate concentration during the memory recall period of a paired associate memory task completed during normoxia. This value compares well with other event-related designs (Mullins, 2018) and is greater in magnitude than that observed in block-related designs (Bednařík et al., 2015; Ip et al., 2017, 2019; Mangia et al., 2012; Martínez-Maestro et al., 2018; Schaller et al., 2014). This leads to our interpretation of the glutamate increase as being partly the result of a compartmental shift in glutamate to a more visible glutamate pool during enhanced neurotransmission (Apšvalka et al., 2015; Mullins, 2018). Thus, prompting the conclusion that the increase in PCC glutamate under normoxic conditions is supporting episodic memory recall during paired associated memory task. However, an alternate hypothesis of this task-induced glutamate increase is available. The smaller increases observed in block-related fMRS designs have been suggested to result from net synthesis of glutamate during enhanced oxidative metabolism (Mangia et al., 2006; Mangia et al., 2012; Schaller et al., 2014). As such, increases in glutamate have the potential to be interpreted as either a direct measure of an increase in neurotransmission, or a measure of increased oxidative metabolism because of increased neurotransmission. We would suggest that any change is likely a weighted average of the two, with the weighted change depending on acquisition scheme settings, such as echo time or paradigm (Mullins, 2018). Again, this hypothesis of task-induced glutamate increase would still support the involvement of the PCC in episodic memory recall during normoxia.

Contrary to our prediction, during hypoxia, our episodic memory recall task did not result in an increase in glutamate concentration within the PCC as was observed in normoxia. Thus, hypoxia negates the PCC glutamate response to episodic memory recall, with a decrease in performance. However, it did not reduce the concentration of glutamate below rest. A reduction in glutamate concentration below resting values has been demonstrated within the same regions that exhibit a negative bold response to a task designed to deactivate a brain region (Bednařík et al., 2015; Martínez-Maestro et al., 2018). This prompts our interpretation that during hypoxia the PCC is no longer responding to the task as it does in normoxia, rather than deactivating or actively suppressing neural activity *per se*. Given that we found episodic memory recall performance was reduced in hypoxia, this could be the result of the loss of functional activation of the PCC. However, it is important to note that although performance was reduced, participants were still performing above chance. This could indicate that there has been a rapid reorganisation in the neural networks that support episodic memory recall, sustaining it in the face of altered PCC functionality (a conclusion suggested by Rossetti et al. (2020)). This interpretation would remain the same, no matter which of the two hypothesised mechanisms behind fMRS changes in glutamate, namely neurotransmission leading to a change in pool visibility or net synthesis with enhanced oxidative metabolism during task induced regional neural activity.

Unexpectedly, when comparing measured metabolite concentrations between normoxia and hypoxia we observed a significant reduction in both the resting and recall concentration of glucose and the recall only concentration of glutathione during hypoxia. Our fMRS acquisition scheme was not optimised for the detection of the low concentration glutathione metabolite, for this reason we will not draw inferences on this specific alteration of this metabolite. Neurovascular coupling usually ensures that glucose supply suffices regional metabolic demand. For this reason, changes in glucose concentration have been used as a surrogate for indexing neural activity such as ^18^FDG-PET imaging methods. Interpreting this finding in terms of hypoxia inducing a reorganisation in the neural networks that support episodic memory recall, this reduction in glucose may reflect the loss of regional CBF increases, thus the delivery of glucose to the PCC is reduced. However, this finding must be considered as preliminary from our 3T PRESS sequence and warrants further investigation using techniques more specific for these metabolites (e.g. edited 1^H^ MRS,13 C MRS or 7T MRS).

However, there is another possible interpretation whereby the balance between oxidative and nonoxidative metabolism supporting functional neural activity within the PCC is altered. Given the hypothesis that glutamate increases detected using fMRS reflect a net synthesis of glutamate due to enhanced oxidative metabolism during neural activity (Mangia et al., 2006, 2012; Schaller et al., 2014), an alteration in the balance of oxidative and nonoxidative metabolism would alter the observed functional glutamate change. Presuming a shift in oxidative metabolism is the origin of the glutamate change we observe in normoxia, we could conclude that the lack of an increase in hypoxia is the result of a shift towards non-oxidative metabolism to sustain neural activity demands within the PCC. The resulting reduction in glucose during hypoxia would reflect its continued consumption by either oxidative or nonoxidative means to meet baseline metabolic demands without compensatory resupply due to the reduction in CBF observed in hypoxia. That is, the reduction seen arises because of reduced delivery that does not match demand, implying that neurovascular coupling is either reversed (as seen in Rossetti et al., 2020), or at the least impaired, in the PCC during hypoxia. As investigating potential glucose changes was not a primary focus of our study, these results and the possible interpretations, while interesting, should be considered speculative, and warrant further investigation at higher fields (7T or above) where measurement of glucose may be more reliable.

The present study has demonstrated that three hours of moderate poikilocapnic hypoxia disrupted episodic memory recall. We are confident that this reduction in performance is a true decrement in episodic memory recall ability rather than a condition specific speed accuracy trade-off or response bias. Few investigations have aimed at understanding the physiological mechanism that underlie hypoxia induced cognitive deficits. An investigation by Williams et al. (2019) revealed that decrements in central executive function were correlated with the degree of peripheral arterial oxygen desaturation and prefrontal cortex deoxygenation. Although in the present study we do not have arterial or cortical oxygenation data to assess the effect of degree of regional hypoxaemia, a disruption of glutamate metabolism, interpreted as an alteration in oxidative metabolism would relate to the conclusions of Williams et al. (2019). However more recently an investigation by Friend et al. (2019) revealed that poikilocapnic hypoxia and hyperventilation hypocapnia resulted in similar decrements in cognitive performance but hypoxaemia (isocapnic hypoxia) alone did not result in a cognitive performance decrement. This implicates hypocapnia and resulting cerebral tissue alkalosis in the development of cognitive impairment in hypoxia. The authors argued that competing vascular constrictive effect of hypocapnia and alterations in the unloading of oxygen at the tissue level may reduce oxygen supply (Friend et al., 2019). In the present study we can estimate that participants were experiencing a mild state of hypocapnia during completion of the memory task. As such we cannot rule out hyperventilation induced hypocapnia as a contributing factor to the hypoxia induced alteration in episodic memory-evoked PCC glutamate metabolism. Future investigations should aim to isolate the hypoxaemia and hyperventilation induced hypocapnia components of poikilocapnic hypoxia on resting and task regional cerebral metabolism and blood flow.

## 5. Conclusions

Hypoxia has been demonstrated to alter normal task-induced changes in glutamate metabolism within the PCC and is suggested to induce a switch to anaerobic metabolism. Reductions in CBF may be reducing oxygen delivery, leading to a slightly anoxic state for the tissue, leading to a switch away from oxidative metabolism, as evidenced by a lack of the normal task-induced glutamate increase. It is likely that this reduction in glutamate represents decreased neural activity in this region to the task during hypoxia, implying that the PCC is no longer operating as part of the neural network that supports episodic memory recall.

## Supplementary Material

1

## Figures and Tables

**Table 1:** Mean Metabolite concentrations at rest for each condition.

	Baseline		
	Normoxia	Hypoxia	% Change

Glutamate	11.19 (2.46)	10.42 (2.43)	−6.87 [−24.35, 10.60]
Glutamine	1.14 (0.91)	0.84 (1.08)	−26.17 [−56.39, 4.04]
Glx(glutamate+glutamine)	12.33 (2.98)	11.26 (2.54)	−8.65 [−25.11, 7.80]
Glucose	0.6 (0.19)	0.56 (0.32)	−5.74 [−62.65, 51.18]
Myo-inositol	5.78 (0.81)	5.66 (1.36)	−2.07 [−18.83, 14.69]
Creatine	14.67 (0.99)	15.08 (1.45)	2.79 [−6.85, 12.42]
Choline	1.32 (0.27)	1.42 (0.37)	7.6 [−10.89, 26.09]
N-acetyl aspartate	7.34 (1.52)	7.55 (1.54)	2.85 [−10.74, 16.44]
Gamma-amniobutyric acid	0.26 (0.69)	0.13 (0.22)	−49.72 [−180.45, 81.01]
Glutathione	1.99 (0.6)	1.68 (0.69)	−15.55 [−41.32, 10.22]

*Note.* Estimated metabolite concertation are shown in millimolar (mM) for each rest condition. Subscripts in brackets below concentration estimation represents the standard deviation of the mean. Percentage change values are calculated as average from the individual change across all participants, relative from normoxia. [95% confidence interval of the mean change].

*Note.* Values represent mean across participants in normoxia and hypoxia. Subscripts in brackets below values represent the standard deviation of the mean. [95% Confidence Interval of mean change]. The D’ prime was calculated from the proportion of hits and false alarms and represents the extent to which task performance deviates from chance (as a Z-score); a higher value of D’ prime is indicative of better performance (from Rossetti et al., 2020).

**Table 2:** Behavioural performance measures on paired associates task in both conditions.

	Normoxia	Hypoxia	% Change from Normoxia	Significance

Accuracy	73.3(8.70)	67.3(8.10)	−8.2[−13, −2]	0.016
Reaction Time	2.64(0.68)	2.74(0.98)	3.5[−7, 14]	0.501
D prime	1.29(0.57)	0.93(0.54)	−27.85[−43, 0.5]	0.011
Beta	1.13(0.39)	1.24(0.50)	9.7[−11, 42]	0.304

**Table 3:** Mean metabolite concentrations (mM) for rest and recall within each condition.

	Normoxia			Hypoxia			Δ% Condition (hypoxia vs normoxia)	
	Rest	Recall	% Change (recall vs rest)	Rest	Recall	% Change (recall vs rest)	Rest	Recall

Glutamate	8.95 (2.91)	9.64 (2.83)	7.72* [0.28, 15.15]	8.18 (1.81)	7.92 (2.05)	−3.22 [−8.86, 2.42]	−8.63 [−19.94, 2.67]	−17.91** [−27.29, −8.54]
Glutamine	0.5 (0.24)	0.5 (0.19)	−0.31 [−53.74, 53.12]	0.34 (0.12)	0.32 (0.14)	−6.51 [−26.37, 13.36]	−32.02 [−137.60, 73.57]	−36.24 [−59.64, −12.84]
Glx (glutamate + glutamine)	9.46 (3.08)	10.15 (2.94)	7.29* [−0.25, 14.83]	8.52 (1.86)	8.24 (2.12)	−3.35 [−8.84, 2.13]	−9.88 [−21.12, 1.36]	−18.82** [−27.74, −9.90]
Myo-inositol	3.25 (0.83)	3.29 (0.71)	1.35 [−3.14, 5.84]	3.01 (1.04)	2.99 (0.94)	−0.67 [−3.50, 2.15]	−7.34 [−21.63, 6.95]	−9.19 [−21.41, 3.04]
Creatine	12.89 (0.94)	12.73 (0.92)	−1.29 [−2.93, 0.36]	12.69 (1.86)	12.58 (2.04)	−0.85 [−3.85, 2.12]	−1.6 [−9.17, 5.97]	−1.17 [−8.81, 6.48]
Choline	1.35 (0.18)	1.36 (0.26)	0.62 [−8.01, 9.25]	1.38 (0.34)	1.38 (0.35)	0.11 [−5.48, 5.70]	1.9 [−12.79, 16.58]	1.38 [−12.77, 15.52]
N-acetyl aspartate	6.44 (1.73)	6.09 (1.56)	−5.45 [−11.92, 1.01]	6.03 (1.43)	5.85 (1.38)	−3.02 [−9.12, 3.09]	−6.4 [−25.52, 12.71]	−3.99 [−24.02, 16.04]
Gamma-amniobutyric acid	0.67 (0.82)	0.61 (0.94)	−9.27 [−60.98, 42.43]	0.45 (0.39)	0.37 (0.41)	−19.02 [−130.64, 92.60]	32.87 [−136.91, 71.17]	−40.08 [−238.46, 158.30]
Glucose	0.7 (0.2)	0.72 (0.21)	3.77 [−7.69, 15.23]	0.57 (0.2)	0.56 (0.23)	−2.02 [−12.39, 8.36]	−17.92* [−34.17, −1.66]	−22.50* [−45.47, 0.48]
Glutathione	0.52 (0.25)	0.52 (0.26)	−.1.24 [−13.65, 11.17]	0.48 (0.23)	0.37 (0.12)	−22.28 [−41.26, −3.30]	−7.96 [−38.43, 22.50]	−27.57* [−46.50, −8.65]

*Note.* Estimated metabolite concentrations are shown in millimolar (mM) for rest and recall within each condition. Subscripts in brackets below concentration estimation represent the standard deviation of the mean. Percentage change values are calculated as average from individual changes across all participants, response relative to rest within conditions (columns 4 and 7), or hypoxia relative to normoxia between condition (columns 8 and 9). [95% confidence interval of the mean change].

* Denotes significance at *P* < .05

** *P* < .001

## Data Availability

Raw data is available from the Open Science Framework project ‘Hypoxia alters posterior cingulate cortex metabolism during a memory task: a 1H fMRS study’: https://osf.io/amsk2/?view_only=6a48f01fafcd496baf6f63c7f122602e

## References

[R1] Ainslie PN, Shaw AD, Smith KJ, Willie CK, Ikeda K, Graham J, Macleod DB, 2014. Stability of cerebral metabolism and substrate availability in humans during hypoxia and hyperoxia. Clin. Sci. (Colch) 126 (9), 661–670. doi:10.1042/CS20130343. 24117382

[R2] Ainslie PN, Subudhi AW, 2014. Cerebral blood flow at high altitude. High Alt. Med. Biol. 15 (2), 133–140. 24971767 10.1089/ham.2013.1138

[R3] Alsop DC, Detre JA, Golay X, Günther M, Hendrikse J, Hernandez-Garcia L, Zaharchuk G, 2015. Recommended implementation of arterial spin-labeled perfusion MRI for clinical applications: a consensus of the ISMRM perfusion study group and the European consortium for ASL in dementia. Magn. Reson. Med. 73 (1), 102–116. 24715426 10.1002/mrm.25197 PMC4190138

[R4] Apšvalka D, Gadie A, Clemence M, Mullins PG, 2015. Event-related dynamics of glutamate and BOLD effects measured using functional magnetic resonance spectroscopy (fMRS) at 3T in a repetition suppression paradigm. Neuroimage 118, 292–300. doi: 10.1016/j.neuroimage.2015.06.015. 26072254

[R5] Bednařík P, Tkáč I, Giove F, Dinuzzo M, Deelchand DK, Emir UE, Mangia S, 2015. Neurochemical and BOLD responses during neuronal activation measured in the human visual cortex at 7 Tesla. J. Cereb. Blood Flow Metab. 35 (October 2014), 601–610. doi:10.1038/jcbfm.2014.233. 25564236 PMC4420878

[R6] Binks AP, Cunningham VJ, Adams L, Banzett RB, 2008. Gray matter blood flow change is unevenly distributed during moderate isocapnic hypoxia in humans. J. Appl. Physiol. 104 (1), 212–217. doi:10.1152/japplphysiol.00069.2007. 17991793

[R7] Boillat Y, Xin L, van der Zwaag W, Gruetter R, 2020. Metabolite concentration changes associated with positive and negative BOLD responses in the human visual cortex: A functional MRS study at 7 Tesla. J. Cereb. Blood Flow Metab. 40 (3), 488–500. doi:10.1177/0271678x19831022. 30755134 PMC7026843

[R8] Brainard DH, 1997. The psychophysics toolbox. Spat Vis; 10, 433–436. 9176952

[R9] Chappell MA, Groves AR, Whitcher B, Woolrich MW, 2008. Variational Bayesian inference for a nonlinear forward model. IEEE Trans. Signal Process. 57 (1), 223–236.

[R10] Duncombe J, Lennen RJ, Jansen MA, Marshall I, Wardlaw JM, Horsburgh K, 2017. Ageing causes prominent neurovascular dysfunction associated with loss of astrocytic contacts and gliosis. Neuropathol. Appl. Neurobiol. 43 (6), 477–491. 28039950 10.1111/nan.12375

[R11] Edden RAE, Harris AD, Murphy K, Evans CJ, Saxena N, Hall JE, Wise RG, 2010. Edited MRS is sensitive to changes in lactate concentration during inspiratory hypoxia. J. Magn. Reson. Imaging 32 (2), 320–325. doi:10.1002/jmri.22233. 20677257

[R12] Friend AT, Balanos GM, Lucas SJ, 2019. Isolating the independent effects of hypoxia and hyperventilation-induced hypocapnia on cerebral haemodynamics and cognitive function. Exp. Physiol. 104 (10), 1482–1493. 31342596 10.1113/EP087602

[R13] Hales PW, Kirkham FJ, Clark CA, 2016. A general model to calculate the spin-lattice (T1) relaxation time of blood, accounting for haematocrit, oxygen saturation and magnetic field strength. J. Cereb. Blood Flow Metab. Off. J. Int. Soc. Cereb. Blood Flow Metab 36 (2), 370–374. doi:10.1177/0271678x15605856. PMC4759664 26661147

[R14] Harris AD, Murphy K, Diaz CM, Saxena N, Hall JE, Liu TT, Wise RG, 2013. Cerebral blood flow response to acute hypoxic hypoxia. NMR Biomed. 26 (12), 1844–1852. 24123253 10.1002/nbm.3026 PMC4114548

[R15] Hochachka PW, Clark CM, Matheson GO, Brown WD, Stone CK, Nickles RJ, Holden JE, 1999. Effects on regional brain metabolism of high-altitude hypoxia: a study of six US marines. Am. J. Physiol. Regul. Integr. Comp. Physiol. 277 (1), R314–R319. doi:10.1152/ajpregu.1999.277.1.r314. 10409288

[R16] Hochachka PW, Clark CM, Monge C, Stanley C, Brown WD, Stone CK, Holden JE, 1996. Sherpa brain glucose metabolism and defense adaptations against chronic hypoxia. J. Appl. Physiol. 81 (3), 1355–1361. doi:10.1152/jappl.1996.81.3.1355. 8889774

[R17] Ip IB, Berrington A, Hess AT, Parker AJ, Emir UE, Bridge H, 2017. Combined fMRI-MRS acquires simultaneous glutamate and BOLD-fMRI signals in the human brain. Neuroimage 155, 113–119. doi:10.1016/j.neuroimage.2017.04.030. 28433623 PMC5519502

[R18] Ip IB, Emir UE, Parker AJ, Campbell J, Bridge H, 2019. Comparison of neurochemical and BOLD signal contrast response functions in the human visual cortex. J. Neurosci. 39 (40), 7968–7975. 31358655 10.1523/JNEUROSCI.3021-18.2019 PMC6774413

[R19] Jensen MLF, Vestergaard MB, Tønnesen P, Larsson HBW, Jennum PJ, 2018. Cerebral blood flow, oxygen metabolism, and lactate during hypoxia in patients with obstructive sleep apnea. Sleep 41 (3), zsy001. 10.1093/sleep/zsy001 29309697

[R20] Kassambara A, 2020. rstatix: pipe-friendly framework for basic statistical tests. R package version 0.6.0. Available from.

[R21] Kozberg MG, Chen BR, DeLeo SE, Bouchard MB, Hillman EM, 2013. Resolving the transition from negative to positive blood oxygen level-dependent responses in the developing brain. Proc. Natl. Acad. Sci. 110 (11), 4380–4385. 23426630 10.1073/pnas.1212785110 PMC3600493

[R22] Kozberg M, & Hillman E (2016). Neurovascular coupling and energy metabolism in the developing brain. 10.1016/bs.pbr.2016.02.002 PMC5134842 27130418

[R23] Kusaka T, Kawada K, Okubo K, Nagano K, Namba M, Okada H, Itoh S, 2004. Noninvasive optical imaging in the visual cortex in young infants. Hum. Brain Mapp. 22 (2), 122–132. 15108300 10.1002/hbm.20020 PMC6871980

[R24] Lally N, Mullins PG, Roberts MV, Price D, Gruber T, Haenschel C, 2014. Glutamatergic correlates of gamma-band oscillatory activity during cognition: A concurrent ER-MRS and EEG study. Neuroimage 85, 823–833. doi:10.1016/j.neuroimage.2013.07.049. 23891885

[R25] Lawley JS, Macdonald JH, Oliver SJ, Mullins PG, 2017. Unexpected reductions in regional cerebral perfusion during prolonged hypoxia. J. Physiol. 595 (3). doi:10.1113/JP272557. PMC5285718 27506309

[R26] Lin A, Andronesi O, Bogner W, Choi I-Y, Coello E, Cudalbu C, Juchem C, Kemp GJ, Kreis R, Krššák M, Lee P, Maudsley AA, Meyerspeer M, Mlynarik V, Near J, Öz G, Peek AL, Puts NA, Ratai EM, Mullins PG, 2021. Minimum reporting standards for in vivo magnetic resonance spectroscopy (MRSinMRS): experts’ consensus recommendations. NMR Biomed. e4484. doi:10.1002/nbm.4484, n/a(n/a). PMC8647919 33559967

[R27] Logothetis NK, 2008. What we can do and what we cannot do with fMRI. Nature 453 (7197), 869–878. doi:10.1038/nature06976. 18548064

[R28] Makowski D, 2018. The psycho package: an efficient and publishing-oriented workflow for psychological science. J. Open Source Softw. 3 (22), 470. https://github.com/neuropsychology/psycho.R.

[R29] Mangia S, Giove F, DiNuzzo M, 2012. Metabolic pathways and activity-dependent modulation of glutamate concentration in the human brain. Neurochem. Res. 37 (11), 2554–2561. doi:10.1007/s11064-012-0848-4. 22846967 PMC3489977

[R30] Mangia S, Tkáč I, Gruetter R, Van De Moortele PF, Giove F, Maraviglia B, Uǧurbil K, 2006. Sensitivity of single-voxel 1H-MRS in investigating the metabolism of the activated human visual cortex at 7 T. Magn. Reson. Imaging 24 (4), 343–348. doi:10.1016/j.mri.2005.12.023. 16677939

[R31] Martínez-Maestro M, Labadie C, Möller HE, 2018. Dynamic metabolic changes in human visual cortex in regions with positive and negative blood oxygenation level-dependent response. J. Cereb. Blood Flow Metab. doi:10.1177/0271678x18795426, 0271678x1879542. PMC6827122 30117749

[R32] Merz TM, Treyer V, Hefti U, Spengler CM, Schwarz U, Buck A, Maggiorini M, 2006. Changes in cerebral glucose metabolism after an expedition to high altitudes. High Alt. Med. Biol. 7 (1), 28–38. 16544964 10.1089/ham.2006.7.28

[R33] Mullins PG (2018). Towards a theory of functional magnetic resonance spectroscopy (fMRS): a meta-analysis and discussion of using MRS to measure changes in neurotransmitters in real time. 10.1111/sjop.12411. 29356002

[R34] Mullins PG, Chen H, Xu J, Caprihan A, Gasparovic C, 2008. Comparative reliability of proton spectroscopy techniques designed to improve detection of J-coupled metabolites. Magn. Reson. Med. 60 (4), 964–969. doi:10.1002/mrm.21696. 18816817

[R35] Naressi A, Couturier C, Devos JM, Janssen M, Mangeat C, de Beer R, Graveron-Demilly D, 2001. jMRUI, MRUI for Java. MAGMA 12, 141–152. 11390270 10.1007/BF02668096

[R36] Nöth U, Kotajima F, Deichmann R, Turner R, Corfield DR, 2008. Mapping of the cerebral vascular response to hypoxia and hypercapnia using quantitative perfusion MRI at 3 T. NMR Biomed. 21 (5), 464–472. doi:10.1002/nbm.1210. 17854023

[R37] Pelli DG, 1997. The VideoToolbox software for visual psycho- physics: transforming numbers into movies. Spat. Vis. 10, 437–442. 9176953

[R38] Roach RC, Hackett PH, Oelz O, Bartsch P, Luks AM, MacInnis MJ, Baillie JK The Lake Louise AMS Score Consensus Committee, 2018. The 2018 lake louise acute mountain sickness score. High Alt. Med. Biol. 19 (1), 4–6. doi:10.1089/ham.2017.0164. 29583031 PMC6191821

[R39] Rossetti GMK, d’Avossa G, Rogan M, Macdonald JH, Oliver SJ, Mullins PG, 2020. Reversal of neurovascular coupling in the default mode network: Evidence from hypoxia. J. Cereb. Blood Flow Metab. doi:10.1177/0271678x20930827. PMC7983511 32538282

[R40] Rossion B, Pourtois G, 2004. Revisiting snodgrass and vanderwart’s object pictorial set: the role of surface detail in basic-level object recognition. Perception 33 (2), 217–236. 15109163 10.1068/p5117

[R41] Schaller B, Xin L, O’Brien K, Magill AW, Gruetter R, 2014. Are glutamate and lactate increases ubiquitous to physiological activation? A 1H functional MR spectroscopy study during motor activation in human brain at 7Tesla. Neuroimage 93 (P1), 138–145. doi:10.1016/j.neuroimage.2014.02.016. 24555953

[R42] Smith SM, 2002. Fast robust automated brain extraction. Hum. Brain Mapp. 17 (3), 143–155. 12391568 10.1002/hbm.10062 PMC6871816

[R43] Smith ZM, Krizay E, Guo J, Shin DD, Scadeng M, Dubowitz DJ, 2013. Sustained high-altitude hypoxia increases cerebral oxygen metabolism. J. Appl. Physiol. 114 (1), 11–18. 23019310 10.1152/japplphysiol.00703.2012 PMC3544513

[R44] Stanley JA, Raz N, 2018. Functional magnetic resonance spectroscopy: the “new” MRS for cognitive neuroscience and psychiatry research. Front. Psychiatry doi:10.3389/fpsyt.2018.00076. PMC5857528 29593585

[R45] Stefan DDCF, Di Cesare F, Andrasescu A, Popa E, Lazariev A, Vescovo E, Graveron-Demilly D, 2009. Quantitation of magnetic resonance spectroscopy signals: the jMRUI software package. Meas. Sci. Technol. 20 (10), 104035.

[R46] Vestergaard MB, Larsson HB, 2019. Cerebral metabolism and vascular reactivity during breath-hold and hypoxic challenge in freedivers and healthy controls. J. Cereb. Blood Flow Metab. 39 (5), 834–848. doi:10.1177/0271678x17737909. 29099292 PMC6498754

[R47] Vestergaard MB, Lindberg U, Aachmann-Andersen NJ, Lisbjerg K, Christensen SJ, Law I, Larsson HBW, 2016. Acute hypoxia increases the cerebral metabolic rate-a magnetic resonance imaging study. J. Cereb. Blood Flow Metab. 36 (6), 1046–1058. doi:10.1177/0271678x15606460. 26661163 PMC4904346

[R48] Wang K, Smith ZM, Buxton RB, Swenson ER, Dubowitz DJ, 2015. Acetazolamide during acute hypoxia improves tissue oxygenation in the human brain. J. Appl. Physiol. 119 (12), 1494–1500. 26472861 10.1152/japplphysiol.00117.2015 PMC4683345

[R49] Wickham H, Averick M, Bryan J, Chang W, D’Agostino McGowan L, François R, Yutani H, 2019. Welcome to the tidyverse. J. Open Source Softw. 4 (43), 1686. doi:10.21105/joss.01686, Available from.

[R50] Williams TB, Corbett J, McMorris T, Young JS, Dicks M, Ando S, Costello JT, 2019. Cognitive performance is associated with cerebral oxygenation and peripheral oxygen saturation, but not plasma catecholamines, during graded normobaric hypoxia. Exp. Physiol. 104 (9), 1384–1397. doi:10.1113/EP087647. 31192502

[R51] Willie CK, Tzeng YC, Fisher JA, Ainslie PN, 2014. Integrative regulation of human brain blood flow. J. Physiol. 592 (5), 841–859. doi:10.1113/jphysiol.2013.268953. 24396059 PMC3948549

[R52] Winkler AM, Ridgway GR, Webster MA, Smith SM, Nichols TE, 2014. Permutation inference for the general linear model. Neuroimage 92, 381–397. 24530839 10.1016/j.neuroimage.2014.01.060 PMC4010955

[R53] Yamada H, Sadato N, Konishi Y, Muramoto S, Kimura K, Tanaka M, Itoh H, 2000. A milestone for normal development of the infantile brain detected by functional MRI. Neurology 55 (2), 218–223. 10908895 10.1212/wnl.55.2.218

[R54] Zhang Y, Brady M, Smith S, 2001. Segmentation of brain MR images through a hidden Markov random field model and the expectation-maximization algorithm. IEEE Trans. Med. Imaging 20 (1), 45–57. 11293691 10.1109/42.906424

