# Supplementary material for: Hypoxia alters posterior cingulate cortex metabolism during a memory task: A 1H fMRS study
Source: Neuroimage. Author manuscript; Available in PMC 2022 Oct 15. (PMC9513808; doi:10.1016/j.neuroimage.2022.119397)
Supplement: 1 [file NIHMS1833286-supplement-1.docx]

Supplementary Materials:

Hypoxia alters posterior cingulate cortex metabolism during a memory task: a 1H fMRS study


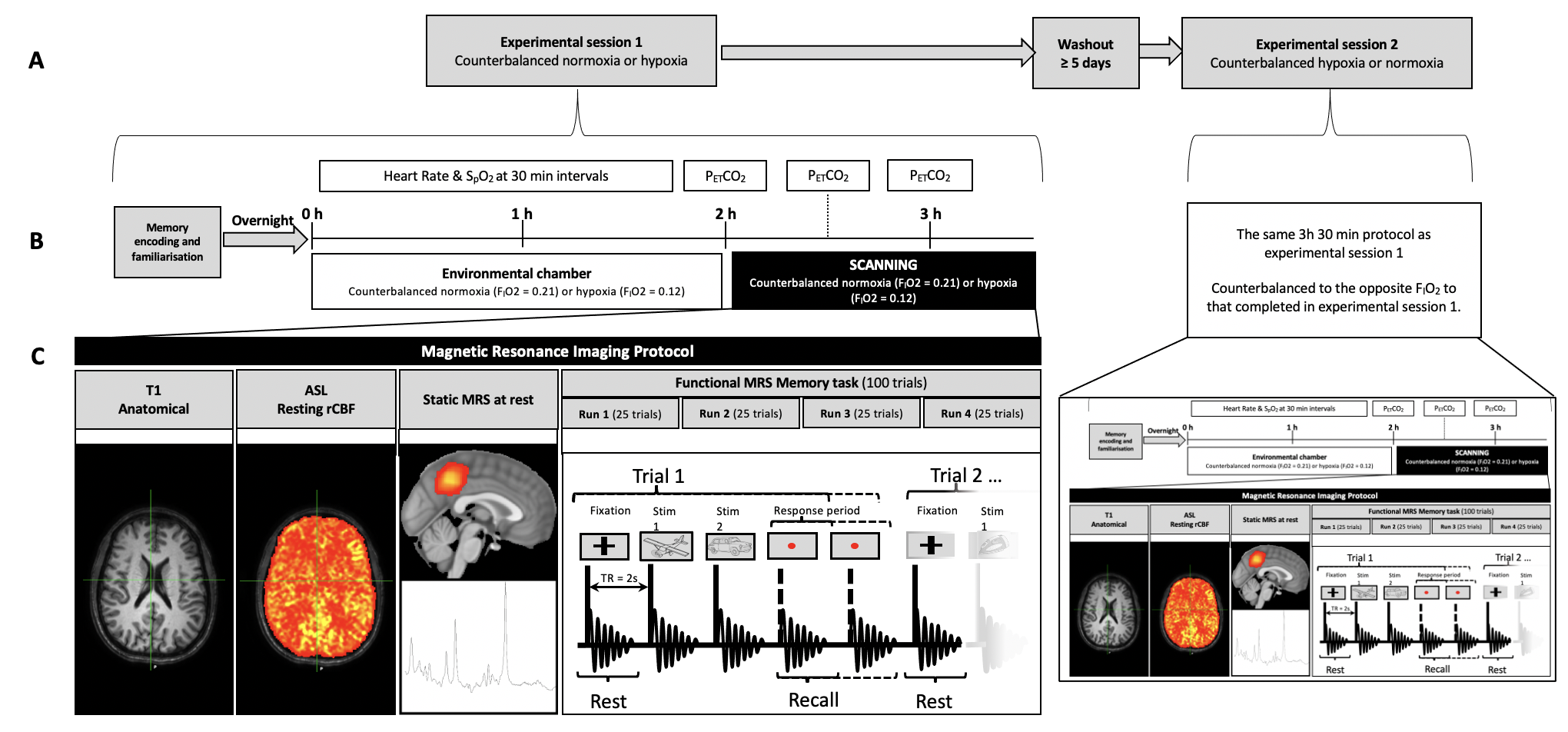


*Supplementary figure S1*. Study design and procedure schematic. Section A displays the counterbalancing of the experimental sessions. Section B displays the within session timeline of events, duration of hypoxic stimulus and the occurrence of data acquisition, both physiological monitoring and MRI scanning. Section C displays the MRI protocol with an example of how spectra acquisition was locked to individual FID acquisition in the functional MRS task.

Supplementary table S1. Physiologic data for each condition

|  | 2 h | | 2.15 h | | 2.30 h | | 3 h | |
| --- | --- | --- | --- | --- | --- | --- | --- | --- |
|  | Normoxia | Hypoxia | Normoxia | Hypoxia | Normoxia | Hypoxia | Normoxia | Hypoxia |
| SpO_2_ | 99  (1) | 84  (7) | - | - | - | - | - | - |
| HR | 68  (8) | 74  (9) | - | - | - | - | - | - |
| P_ET_CO_2_ | - | - | 35  (5) | 33  (6) | 36  (8) | 31  (7) | 35  (7) | 30  (3) |

*Note.* Condition comparison of physiology data. Peripheral arterial oxygen saturation (SpO_2_), Heart Rate (HR) and Partial pressure of end tidal carbon dioxide ( P_ET_CO_2_). Values in () represent the standard deviation of the above mean value.

Supplementary table S2. Physiologic data and calculated blood T1 values for ASL analysis

| Participant Number | Normoxia | | | Hypoxia | | |
| --- | --- | --- | --- | --- | --- | --- |
|  | SpO_2_ | Haematocrit | T1 Blood | SpO_2_ | Haematocrit | T1 Blood |
| 1 | 100 | 47.7 | 1.70 | 85 | 43.0 | 1.64 |
| 2 | 98 | 44.3 | 1.73 | 87 | 47.7 | 1.58 |
| 3 | 100 | 37.3 | 1.83 | 87 | 36.0 | 1.76 |
| 4 | 99 | 43.0 | 1.75 | 96 | 40.3 | 1.73 |
| 5 | 100 | 37.7 | 1.83 | 76 | 40.3 | 1.65 |
| 6 | 98 | 48.3 | 1.68 | 91 | 46.7 | 1.62 |
| 7 | 100 | 38.7 | 1.81 | 89 | 37.7 | 1.74 |
| 8 | 97 | 46.3 | 1.70 | 88 | 42.3 | 1.67 |
| 9 | 100 | 46.7 | 1.71 | 78 | 46.7 | 1.56 |
| 10 | 100 | 40.3 | 1.79 | 92 | 40 | 1.72 |
| 11 | 100 | 48.0 | 1.70 | 92 | 48.7 | 1.60 |
| 12 | 99 | 47.0 | 1.70 | 83 | 44.0 | 1.62 |
| 13 | 100 | 43.3 | 1.75 | 79 | 44.0 | 1.60 |

*Note.* SpO_2_ is peripheral arterial oxygen saturations measured using pulse oximetry at the 2 h time point in each condition. Haematocrit was estimated using a capillary finger-tip blood sample taken at the start of each session. Both of these values were then used in the model suggested by Hales et al., 2016 to estimated individual T1 values for each participant.


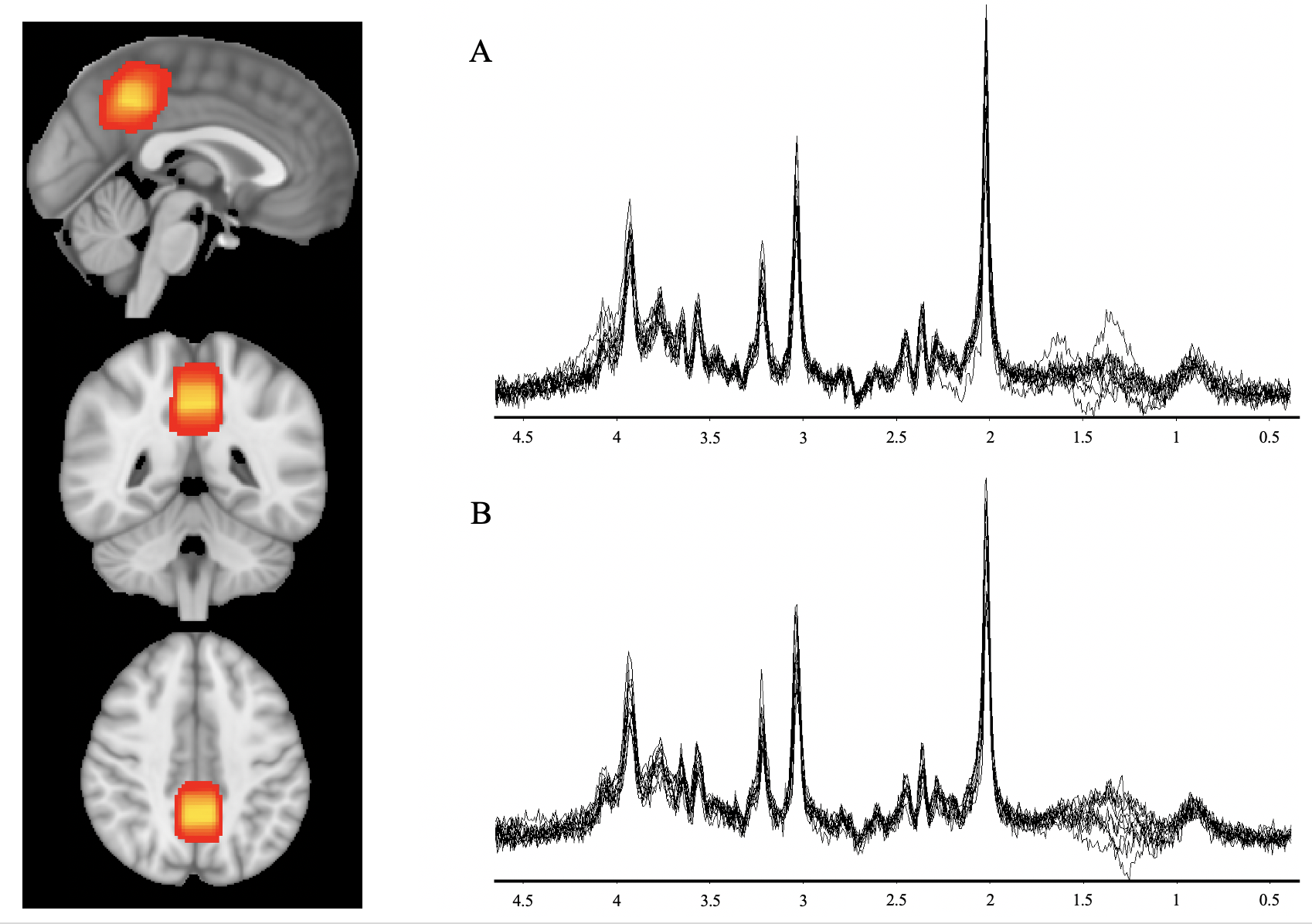


*Supplementary figure 2*. The average location of the MRS acquisition voxel is shown on the left. Yellow reflects greater overlap in positioning across participants and conditions. Acquired spectra across all participants in each condition are shown for visual assessment of quality.


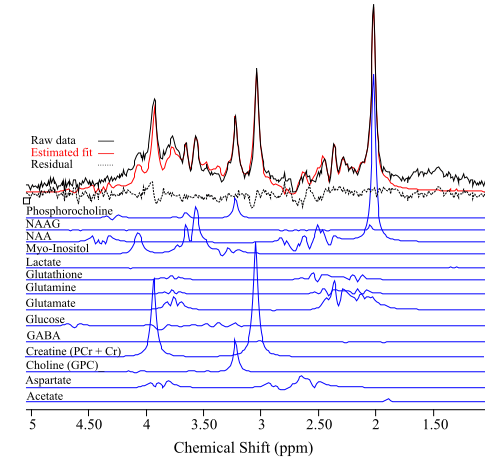


*Supplementary Figure S3.* Estimated fit (in red) residual (Black dashed line) and estimated components of fit (in Blue) from one participant during the "rest" period of the fMRS task in normoxia.
